# Cell osteogenic bioactivity mediated precisely by varying scaled micro-pits on ordered micro/nano hierarchical structures of titanium

**DOI:** 10.1093/rb/rbac046

**Published:** 2022-07-01

**Authors:** Yanmei Zhang, Xiankuan Wang, Yaxian Li, Jianhe Liang, Pinliang Jiang, Qiaoling Huang, Yun Yang, Hongping Duan, Xiang Dong, Gang Rui, Changjian Lin

**Affiliations:** State Key Laboratory of Physical Chemistry of Solid Surfaces, College of Chemistry and Chemical Engineering, Xiamen University, Xiamen 361005, China; State Key Laboratory of Physical Chemistry of Solid Surfaces, College of Chemistry and Chemical Engineering, Xiamen University, Xiamen 361005, China; State Key Laboratory of Physical Chemistry of Solid Surfaces, College of Chemistry and Chemical Engineering, Xiamen University, Xiamen 361005, China; State Key Laboratory of Physical Chemistry of Solid Surfaces, College of Chemistry and Chemical Engineering, Xiamen University, Xiamen 361005, China; State Key Laboratory of Physical Chemistry of Solid Surfaces, College of Chemistry and Chemical Engineering, Xiamen University, Xiamen 361005, China; Research Institute for Biomimetics and Soft Matter, Fujian Provincial Key Laboratory for Soft Functional Materials Research, College of Physical Science and Technology, Xiamen University, Xiamen 361005, China; Research Institute for Biomimetics and Soft Matter, Fujian Provincial Key Laboratory for Soft Functional Materials Research, College of Physical Science and Technology, Xiamen University, Xiamen 361005, China; Beijing Engineering Laboratory of Functional Medical Materials and Devices, Beijing Medical Implant Engineering Research Center, Beijing Naton Technology Group Co. Ltd, Beijing, China; Beijing Engineering Laboratory of Functional Medical Materials and Devices, Beijing Medical Implant Engineering Research Center, Beijing Naton Technology Group Co. Ltd, Beijing, China; Department of Orthopedics Surgery, The First Affiliated Hospital of Xiamen University, Xiamen, Fujian 361003, China; State Key Laboratory of Physical Chemistry of Solid Surfaces, College of Chemistry and Chemical Engineering, Xiamen University, Xiamen 361005, China; Tan Kah Kee Innovation Laboratory, Xiamen, Fujian 361005, China

**Keywords:** electrochemical self-organizing etching, varying micro-scaled pits, osteogenic, Wnt/β-catenin

## Abstract

Hierarchical surface structures with micro–nano scale play a crucial role in regulation of cell proliferation and osteogenic differentiation. It has been proven that cells are extremely sensitive to the nanoscaled structure and show multifarious phenotypes. Though a vital function of microstructure on osseointegration has been confirmed, the cell performances response to different microscaled structure is needed to be further dissected and in depth understood. In this work, the ordered micro–nano hierarchical structures with varying micro-scaled pits were precisely fabricated on titanium successfully by the combination of electrochemical, chemical etching and anodization as well. *In vitro* systematical assessments indicated that the micro–nano multilevel structures on titanium exhibited excellent cells adhesion and spreading ability, as well as steerable proliferation and osteogenic differentiation behaviors. It is shown that smaller micro-pits and lower roughness of the hierarchical structures enabled faster cell propagation. Despite cell growth was delayed on micro–nano titanium with relatively larger cell-match-size micro-pits and roughness, osteogenic-specific genes were significantly elevated. Furthermore, the alkaline phosphatase activity, collagen secretion and extracellular matrix mineralization of MC3T3-E1 on multi-scaled titanium were suppressed by a large margin after adding IWP-2 (an inhibitor of Wnt/β-catenin signal pathway), indicating this pathway played a crucial part in cell osteogenic differentiation modulated by micro–nano structures.

## Graphical Abstract

**Figure:**
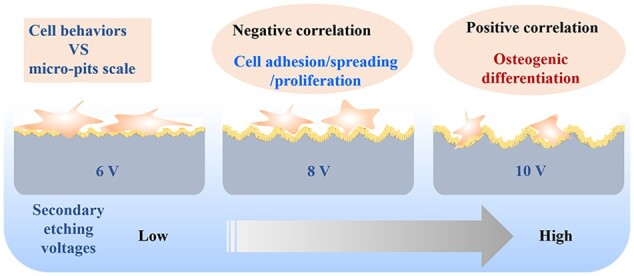

## Introduction

Surface–interface of biomaterials, where cells interact with materials, always plays a crucial role in regulating cell behaviors and determining cell fates. A satisfactory biocompatibility is considered as the criterion need to be complied for materials in medical applications. As the first generation of biomaterials, titanium and its alloys have been widely recognized for orthopedics and dental implants on the strength of non-toxic, light-weight, excellent mechanical property, corrosion resistance and biocompatibility [1, 2]. However, compared with bioactive ceramics, bio-glasses and hydroxyapatite, etc., some intrinsic characteristics of titanium such as bio-inertness, weak osteo-genesis and osteo-induction have become the critical factors hampering bioactive reaction between titanium implants and surrounding tissues [3, 4]. Therefore, the researchers have made extensive studies to develop variety of approaches to improve bioactivity, cell proliferation and osteogenic differentiation abilities of titanium implant surface.

Among them, the regulation of topological morphologies on cell behaviors has attracted more attentions. The cell viability and other biological characteristics could be modulated or improved through the micro-, nano- and micro/nano-hierarchical structures on titanium. It has been suggested that micron-roughed surfaces are beneficial to osteoblasts differentiation, which could induce faster bone maturation and offer better osseointegration. However, it has been found that obvious decrease of cell population on microstructural surface leads to less bone mass [5–10]. Although nanostructure on titanium plays a positive role on promoting cell adhesion, propagation and differentiation, low roughness of pure nano-structure would give rise to insufficient mechanical locking capability, which is not conducive to binding with bone tissue [11–13]. From this perspective, various micro–nano multilevel structures were constructed on titanium to integrate advantages of micro and nano morphology. More importantly, many *in vivo* results verified that implants with micro–nano structures could induce more new bone formation and tighter bond with surrounding tissues [14–21].

Cells were extremely sensitive to subtle variation of implant surface, especially for topological morphologies. The biological performances such as cell adhesion, spreading, migration, proliferation, differentiation, production and calcification of extracellular matrix (ECM), etc. could be significantly impacted by the size, shape and variation scope of surface micro/nano structure [22–27]. It has been reported frequently that the cells fate could be altered by nanoscale changes on titanium [28, 29]. Kubo *et al.* [30] indicated that nano-nodules with the diameter of 300 nm on sandblasting and large-grits etching (SLA) titanium prepared via sputtering deposition offered more favorable microenvironment for osteoblasts than 100 nm or 500 nm. Furthermore, TiO_2_ nanotubes with larger diameter of 100 nm were demonstrated to increase the expression of osteogenic-specific genes hugely compared to 30 nm [14]. Undoubtedly, cell biological behaviors influenced by different nano-scaled structures are diversified.

In addition, microstructure also played non-negligible role on directing cell osteogenic differentiation. However, the effects of microscale structural differences on the cellular biological properties still need to be further studied. Micro-roughed structures on titanium surface can be prepared by chemical etching [30, 31], micro arc oxidation [32, 33], electrochemical etching [34], femtosecond laser processing [35] and so on. It should be noted that the microstructures constructed by chemical etching or micro arc oxidation are usually irregular and uncontrollable. Moreover, plasma etching and femtosecond laser processing are costly and time consuming. By contrast, electrochemical methods not only require quite simple equipment and are easy to operate, but also are able to precisely construct the microstructures with controllable size and shape through adjusting the voltage, etching time and electrolyte conditions. Zinger *et al*. [36] fabricated spherical hollow micron structures on titanium by means of electrochemical processing, and found MG63 cells could enter, adhere and propagate into the micro-holes with the diameter of 30 μm or 100 μm, while could not recognize the micro-cavity with the diameter of 10 μm. Systematic investigation on cell osteogenic differentiation regulated by microscale structure and its corresponding mechanism are still scarce. In this work, an ordered micro–nano hierarchical structure with controllable micro-size was precisely constructed on titanium for the first time through the combination of electrochemical, chemical etching and anodization as well. The osteogenic bioactivity including cell growth, differentiation and related signal pathway mediated by the ordered micro–nano hierarchical structure were analyzed and discussed thoroughly.

## Materials and methods

### Sample preparation

**Figure 1.**
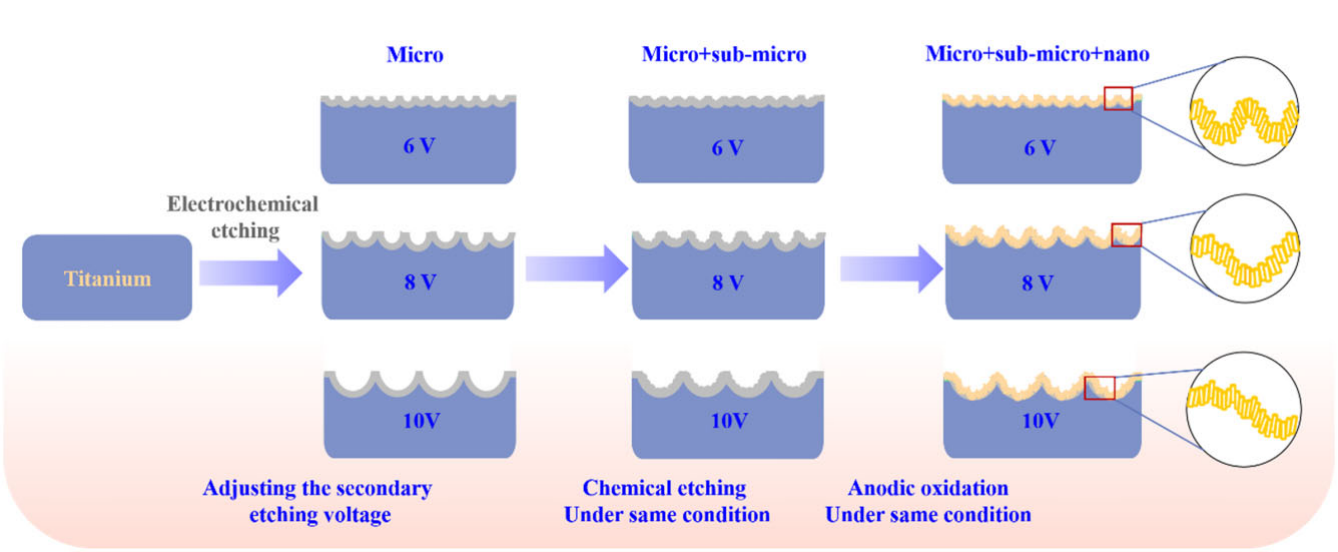
Schematic diagram of preparation process of ordered micro–submicro–nano hierarchical structures with varying scaled micro-pits on titanium.

The preparation process of ordered micro–submicro–nano hierarchical structures with varying scaled micro-pits was illustrated in Fig. 1. Titanium wafers (φ = 15 mm) were abraded carefully using silicon carbide sandpaper, then ultrasonically cleaned in acetone, ethanol and deionized water successively. After that, polished titanium was etched in electrolyte consist of 0.95 M NaCl and 1.2 M HF at 10 V for 10 min via electrochemical reaction, then etched for another 20 min by adjusting the voltage to 6 V, 7 V, 8 V, 9 V or 10 V to make micro-pits array with different size on titanium. After that, the samples were placed into a mixed solution of sulfuric acid and hydrochloric acid (48 *wt*% H_2_SO_4_: 18 *wt*% HCl = 1:1) and reacted at 80°C for 15 min to form sub-micro protuberances array inside each micro-pits, followed by ultrasonic treatment to remove the acid residue on the surface. The treated samples were then electrochemically anodized in aqueous hydrofluoric acid (0.5 *wt*%) for 15 min at 5 V, by counter electrode of a platinum plate, to superimpose nanopores array of titanium dioxide on the structure with micro-pits/submicro-protuberances array. The prepared ordered micro–submicro–nano hierarchical titanium was annotated as 6 V, 8 V and 10 V based on the secondary electrochemical etching voltage, corresponding to different size of TiO_2_ micro-pits, respectively.

### Surface properties

The surface morphology and element content of each sample was observed and analyzed by field emission scanning electron microscope (FE-SEM S4800, Hitachi, Japan) equipped with energy dispersive spectrometer at voltage of 15 KV. The surface roughness was measured by laser scanning microscope (KeyenceVK-X200K, Japan). In addition, surface wettability was examined by dynamic contact angle measuring instrument (Dataphysics OCA20, Germany). BCA protein concentration detection Kit was used to analyze the adsorbing capacity of human serum albumin (HSA) adsorbed on micro–nano hierarchical titanium.

### Cell adhesion and proliferation

Mouse pre-osteoblasts (MC3T3-E1) were cultured in minimum Eagle’s medium alpha modification (α-MEM, with L-glutamine, with ribo- and deoxyribonucleosides) contained 10% fetal bovine serum (FBS) and 1% penicillin–streptomycin in 5% carbon dioxide incubator at 37°C. Upon to 80% confluence, adherent cells were digested by trypsin and diluted to 2 × 10^4^ cells/ml, then they were seeded on the sterilized as-prepared samples. After incubation for 5 h and 24 h, the non-adherent cells were washed with PBS for three times gently, then fixed in 2.5% glutaraldehyde solution. Followed by PBS rinsing, cytoskeletal actin and cell nucleus were stained by phalloidin–tetramethylrhodamine B isothiocyanate (Sigma-Aldrich) and 4′,6-diamidino-2-phenylindole, respectively. Then laser scanning confocal microscopy (CLSM, Leica SP8-STED 3X, Germany) was applied to record the staining results. The live cells on each sample were dyed by calcein-AM and observed under fluorescence microscope (Olympus IX73, Japan). Water-soluble tetrazolium (WST-1) assay was carried out to evaluate the cell proliferation after cultured on each sample for 1, 4 and 7 days. Correspondingly, cells morphologies were examined by FE-SEM after immobilized in 2.5% glutaraldehyde solution, dehydrated via gradient concentration of ethanol, freeze drying and spraying platinum, successively.

### Osteoblast differentiation

Pre-osteoblasts were cultured on the as-prepared micro–submicro–nano titanium at the density of 1.5 × 10^4^ cells/ml. After 4 and 7 days of incubation, the activity of alkaline phosphatase (ALP) was evaluated using ALP detection kit (Beyotime, China) following the manufacturer’s protocol and normalized by the total protein content. Calcium nodule or collagen was stained by alizarin red solution (40 mM, pH = 4.2) or Sirius red (0.1%, dissolved in saturated picric acid) after culturing for 10 days, respectively. The dyeing results were observed under 3D video microscope (Leica DVM 6, Germany). Subsequently, 10% hexadecylpyridinium chloride or mixture solution of 0.2 M NaOH and methyl alcohol (1:1) was introduced to elute the dye. Semi-quantitative analysis of ECM mineralization or collagen secretion was realized via absorbance detection of eluent at 562 nm or 515 nm separately.

### Osteogenic gene expression

MC3T3-E1 cells of 2 × 10^4^ cells/ml were seeded on the as-prepared micro–submicro–nano titanium (φ = 20 mm) and cultured for 4, 7 and 10 days. At each point, total RNA of each sample was isolated by means of Trizol method. Followed by, the process of mRNA reverse transcription was carried out at 37°C for 15 min and 98°C for 5 min. Real-time fluorescence quantification polymerase chain reaction (RT qPCR) was proceeded to assess expression of osteogenic-specific genes including integrin (*ITG*), Runt-related transcription factor 2 (*RUNX-2*), *ALP*, osteocalcin (*OCN*), osteopontin (*OPN*), collagen I (*Col-1*), which normalized by housekeeping gene *GAPDH* according to instruction of 2× SYBR green qPCR mater mix kit (Bioss Antibodies, China). The primer sequences in this study are listed in Supplementary Table S1.

### Wnt/β-catenin inhibitor IWP-2 treatment

MC3T3-E1 cells were seeded on micro–submicro–nano structured titanium (φ = 15 mm) with varying micro-scaled pits at the density of 10,000 cells/sample and pre-cultured for 24 h, then IWP-2 as Wnt/β-catenin signal pathway inhibitor at the final concentration of 1μΜ was added into the culture medium. After 10 days incubation, BCIP/NBT alkaline phosphatase color development kit (Beyotime, China) was applied to detect the ALP activity according to operating instructions. Meanwhile, collagen secretion and ECM mineralization were stained and semi-quantitated following the procedure in Section ‘Osteoblast differentiation’.

### Statistical analysis

The data were presented as average ± standard deviation. All tests were repeated at least three times, and more than three duplicate samples were used in all experiments. One-way analysis of variance was applied to evaluate the statistical differences between two sets of data. Generally, it was considered that there was a significant difference or extremely significant difference in statistics between two sets of data when *P* < 0.05 or *P* < 0.01, which labeled as * or **, respectively.

## Results and discussion

### The morphology and physicochemical properties

**Figure 2.**
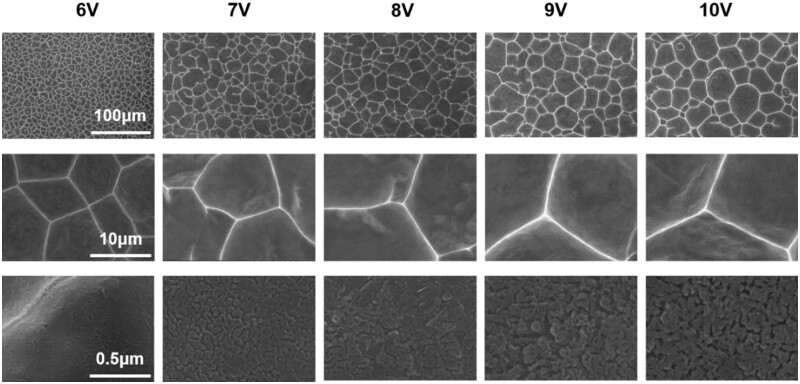
Morphology of varying micro-scale pits array on titanium upon different etching voltages.

**Figure 3.**
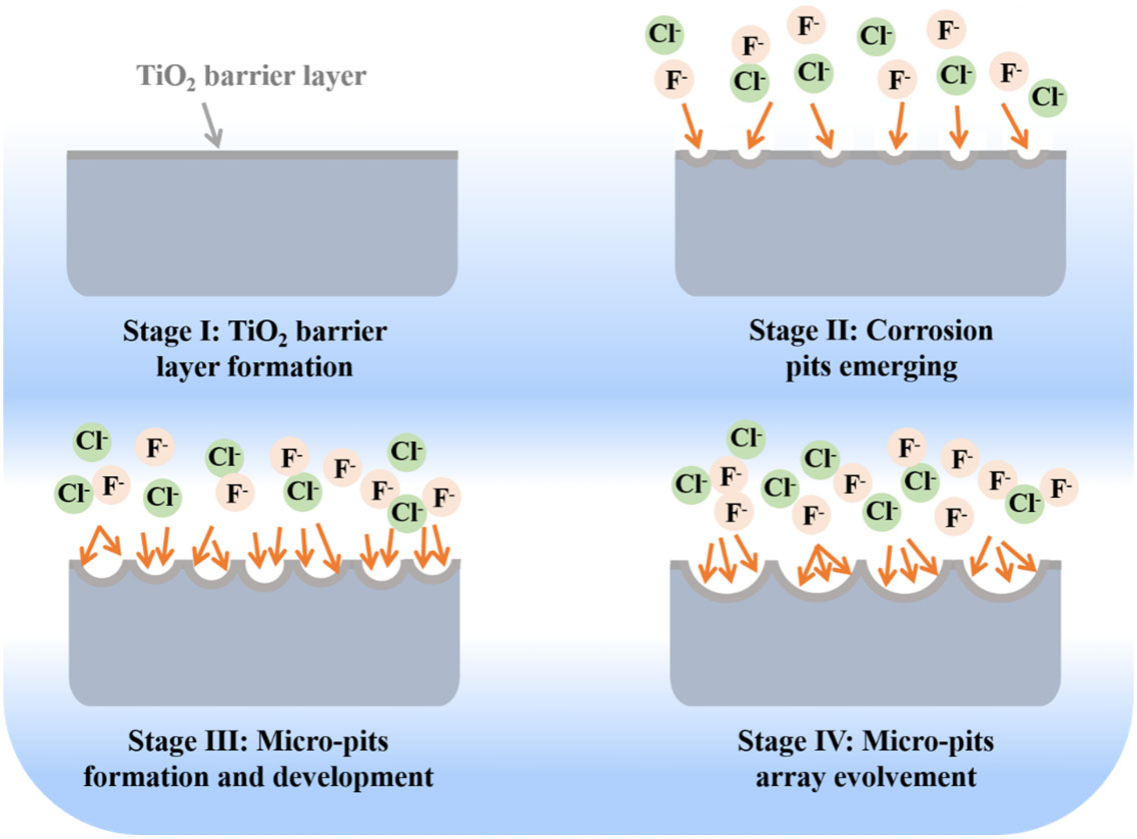
Schematic diagram of the formation process of micro-pits array on titanium.

As shown in Fig. 2, micro-pits array with different size on titanium were controllably fabricated by adjusting the secondary etching voltages. Regular micro-pits array in the shape of polygon were observed clearly on all titanium samples, and the size of micro-pits increased gradually as the voltage rising from 6 V to 10 V. Statistics results as shown in Supplementary Fig. S1 revealed that the average diameter of micro-pit was ∼12.01 μm, 21.36 μm, 23.64 μm, 28.88 μm and 36.34 μm at the secondary voltage of 6 V, 7 V, 8 V, 9 V and 10 V, respectively. The schematic diagram of formation of micro-pits array on titanium was visualized in Fig. 3. The whole process involved four stages and was summarized as follows. Stage I—TiO_2_ barrier layer formation: a certain thick titanium dioxide film (TiO_2_) was formed rapidly within several or dozens of seconds on titanium anode upon powered up and the currents dropping sharply. Stage II—micron pits array emerging: followed by the anode current increased slightly, uniformly dispersed micro-pores emerged through the localized corrosion reaction of TiO_2_ films assisted by fluoride ions as well as chloride ions. Stage III—micro-pits formation and development: then the synergistic action of more F^–^ and Cl^–^ enlarged the size of pre-generated micro-pores, the diameter and number of micro-pits escalated. Adjoining micro-pits began to contact with each other and competitive etching occurred. Stage IV—micro-pits array evolvement: when the competitive etching was extended to the entire Ti electrode surface, the monodisperse micro-pits were transformed into micron pits array under the self-organizing etching by electrochemical field-assisted mechanism. As etching went on, the anode current maintained at a relatively constant value but a large fluctuation which owing to the fusion of adjacent micro-pits [37]. The local current enhanced with an increase of secondary voltages, and the size of micro-pits expanded gradually. The positive correlation between the micro-pits size and secondary voltage was similar to the phenomenon that the diameters of nanotubes were always determined by final anodic voltage, already reported in our previous publication [29]. From the high magnification images of SEM, it can be seen that there were some fold fractures inside the micro-pits. The higher voltages, the larger folds were found, probably due to the non-uniform precipitates of corrosion products inside the micro-pits during etching process.

The CLSM was used to detect the three-dimensional morphologies and roughness of titanium with varying scaled micro-pits (Supplementary Fig. S2). The surface of pristine titanium was smooth, the Ra and Rq was ∼1.704 μm and ∼2.252 μm, respectively. After electrochemically etched, the roughness was increased obviously, Ra and Rq were elevated to ∼2.187 μm and 2.830 μm at secondary voltage of 6 V. When the secondary etching voltage raised to 7 V, 8 V or 9 V, no significant difference of roughness was detected, and the value of Ra maintained in the range of 2.3–2.4 μm. While Ra was slightly increased to ∼2.687 μm when the applied secondary voltage was consistent with the first voltage. The overall trend of roughness was as follows, Ra_(10V)_>Ra_(7V)_≈Ra_(8V)_≈ Ra_(9V)_>Ra_(6V)_>Ra_(Ti)_. Therefore, the micro-pits with smaller size and relative regularity were obtained by using lower voltage (such as 6 V) and suitable etching time. With the secondary voltage increased, the micro-pits began to merge to each other and evolved to larger micro-pits, the surface pits became irregular and the uniformity decreased. Thus there was almost no change in roughness for 7 V, 8 V and 9 V. As the secondary voltage increased further, the size of micro-pits extended and kept stability at a certain extent, while deeper micro-pits and more roughness presented on titanium due to the larger local current as shown in Supplementary Table S2.

**Figure 4.**
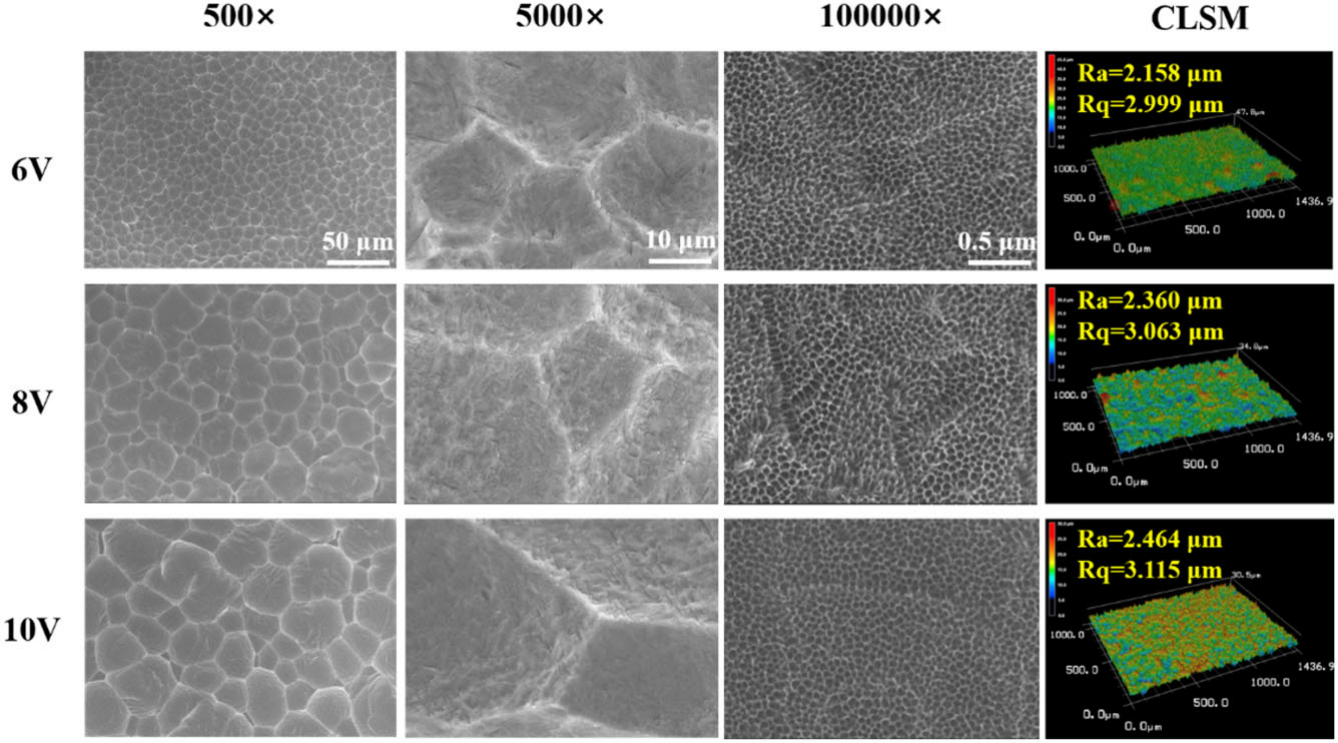
SEM and CLSM results of the prepared ordered micro–nano hierarchical titanium with varying micro-scaled pits.

It has been reported that the surface of biomaterials with only microstructure is not conducive to cell growth [5, 6, 30]. In order to improve the cell biological performance, the sub-micro and nano structures inside the micro-pits were built by the follow-up chemically etching and electrochemically anodizing, without altering original morphologies and sizes of the micro-pits. Consequently, the folds like cracks inside the micro-pits were replaced by submicron protrusions and aligned nanopores. Finally, the ordered micro–submicro–nano hierarchical structures with varying scaled micro-pits were successively fabricated on titanium by adjusting the secondary etching voltages. Compared to single microstructure, the overall roughness was reduced, the gradually increasing trend of roughness was maintained with the increase of secondary voltages (Fig. 4). The average Ra of 6 V, 8 V and 10 V treatment was ∼2.158 μm, ∼2.360 μm and ∼2.464 μm, the Rq was ∼2.999 μm, ∼3.063 μm and ∼3.115 μm, respectively. Chemical etching and anodization preferentially acted on the edges of micro-pits during the etching process, which could be the main reason for the reduction of the surface roughness.

The element analysis of the as-prepared micro/nano hierarchical titanium samples are listed in Table 1. As for pristine titanium, the major elements were Ti and O due to spontaneous formation of titanium oxide in ambient condition. The oxygen concentration of micro/nano structured titanium was enhanced because of the thicken oxide film. The fluoride ions in electrolyte could lead to a small amount of F remains, and the major elements of O, F and Ti kept no significant difference at different etching conditions.

**Table 1.** Element analysis of the prepared micro–nano hierarchical titanium with varying micro-scaled pits

	Ti	6V	7V	8V
**O**	4.75	16.76	16.64	17.02
**F**	N.D.	9.51	9.89	9.86
**Ti**	95.25	73.73	73.47	73.12

**Figure 5.**
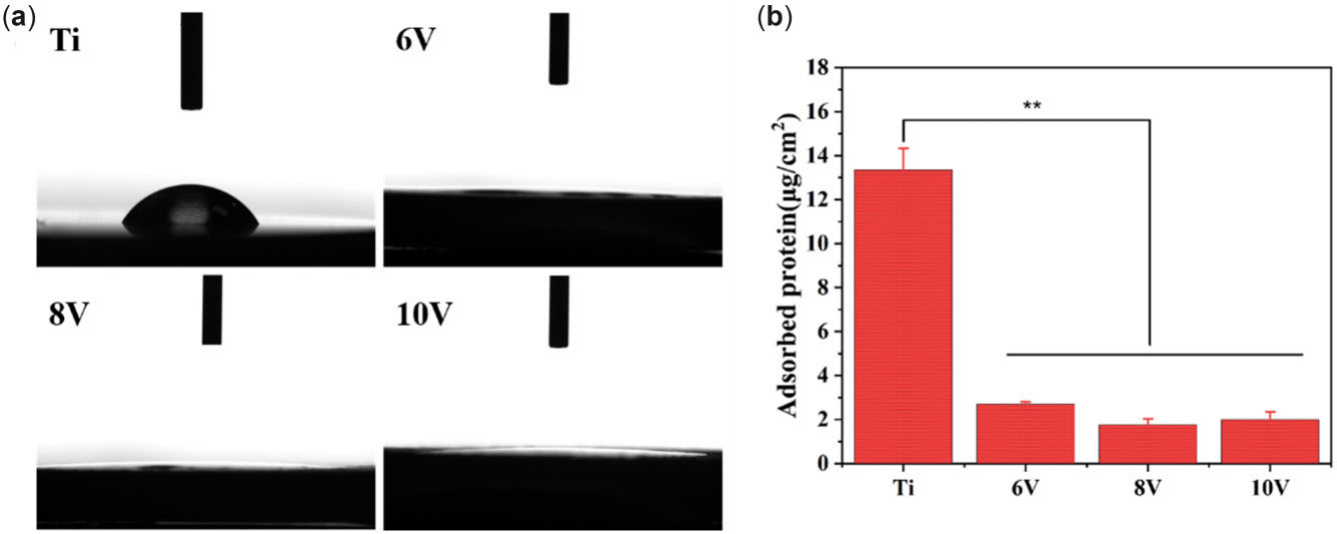
The results of static contact angles (**a**) and protein adsorption (**b**) on the as-prepared micro–nano hierarchical titanium. **P < 0.01.

The results of static water contact angles for the micro–nano hierarchical titanium were shown in Fig. 5a. The contact angle of pristine Ti was 61.2 ± 2.9°, while all contact angles on the prepared micro–nano hierarchical structures were below 5°, showing super-hydrophilic property. After treated with electrochemical, chemical etching and anodization process, the micro-pits and porous nano TiO_2_ films were formed on titanium, which was strongly prone to adsorb H_2_O molecules and exhibited hydrophilic property [16, 37]. Meanwhile, increased roughness could provide more contact area and greater surface tension, so that the surface wettability was further improved [38, 39].

The amount, type and conformation of proteins adsorbed on surface usually have a significant impact on the following biological behaviors of cells. The adsorption capacity of protein is strongly influenced by surface topological morphology, chemical composition, surface charge, etc. [40, 41]. In this work, the protein adsorption capacity on the ordered micro–nano hierarchical titanium was tested by BCA protein concentration detection kit. Surprisingly, the protein adsorbed on pristine Ti surface appeared highest, while the protein amount decreased dramatically on the 6 V, 8 V and 10 V treated samples (Fig. 5b). Generally speaking, higher wettability meant greater surface energy which could promote early cellular responses like cell adhesion and spreading at the interface of implants via adsorb more proteins [42]. Although micro–nano hierarchical titanium presented higher roughness, larger specific surface area and super-hydrophilicity compared with pristine titanium, less HSA adsorption was observed. It was suggested that hydrophobic interaction may play a vital role compared with hydrogen bonding, van der Waals forces and electrostatic interaction in the adsorption process of protein on the material surface. The prepared micro/nanostructure with super-hydrophilicity could weaken the hydrophobic interaction and the H_2_O molecules would race to the surface faster than protein [43]. Thus, the amount of protein adsorption on the hierarchically structured surface was greatly reduced. As for the different size of micro/nano hierarchical structures, it had little effect on the protein adsorption because of the strongly preferential adsorption of H_2_O molecules on the titanium surface.

### Cell adhesion and proliferation

**Figure 6.**
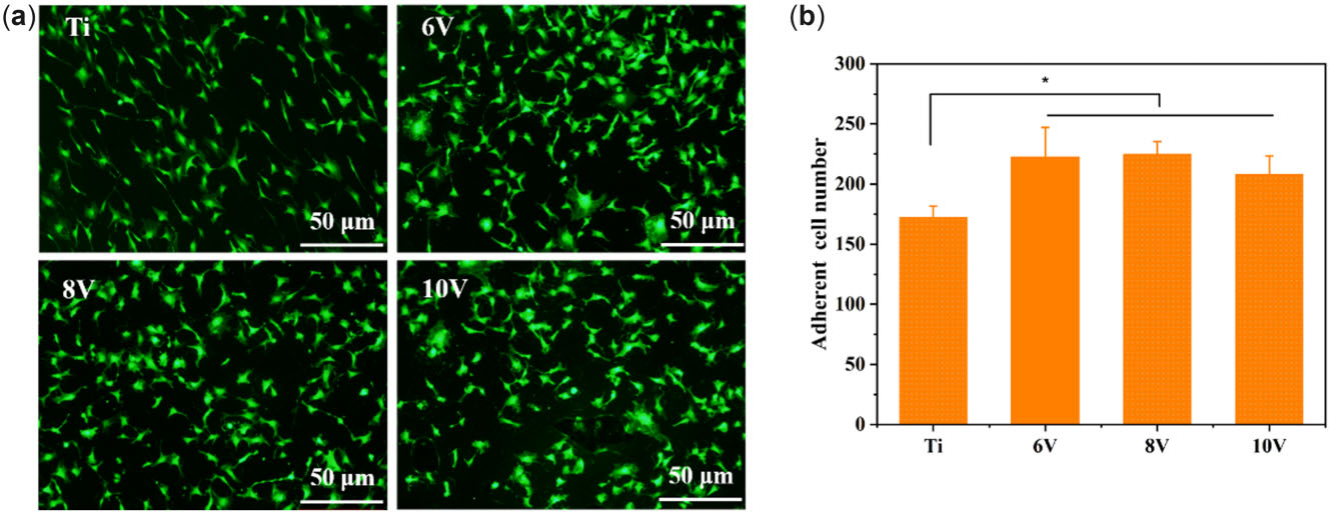
The results of cell fluorescence staining (**a**) and histogram of cell adhesion population (**b**) on hierarchical titanium with varying micro-scaled pits. *P < 0.05.

The results of Calcein-AM fluorescence staining shown in Fig. 6a indicated that MC3T3-E1 cells possessed brilliant adhesion behavior on all the as-prepared titanium after 24-h incubation. On the whole, the number of cell adhesion on the surface of micro/nano structured titanium was higher than that on pristine titanium, and there was significant difference. It was suggested that the hierarchical structures with ordered micro, submicro and nano morphology were beneficial to cell adhesion at the early stage, but this behavior was not sensitive to the micro-scaled changes. Moreover, the phenomenon that similar amount of adhered cells on uniform nano-morphology proved the importance of nanostructure on cell adhesion [13]. As can be seen from Fig. 6b, larger amount of cells adhered to the surface of 6 V or 8 V treated samples, and a little less cells adhered to 10 V titanium. Except for nano-morphology, roughness also played a critical role on cell adhesion. Cells did not tend to adhere to the surface with more roughness according to the works in literatures [35]. This probably gave a reasonable explanation for less cell adhesion was observed on 10 V samples. Cell adhesion and proliferation could be influenced by protein adsorption. As seen in Fig. 5b, the amount of HSA on micro/nanostructures decreased dramatically due to the surface super-hydrophilicity. Interestingly, increased number of cells were observed on the ordered micro/nano hierarchical titanium because of its larger specific surface area and higher wettability [44]. Huang *et al.* [45] indicated that different cells had discrepant adhesion behaviors on whether super-hydrophilic or super-hydrophobic surface. And presence or absence of FBS showed little effect on the cell adhesion. Therefore, it was speculated that, as for MC3T3-E1, the nano-morphology or hydrophilicity played more crucial role in their adhesion than the protein adsorption.

**Figure 7.**
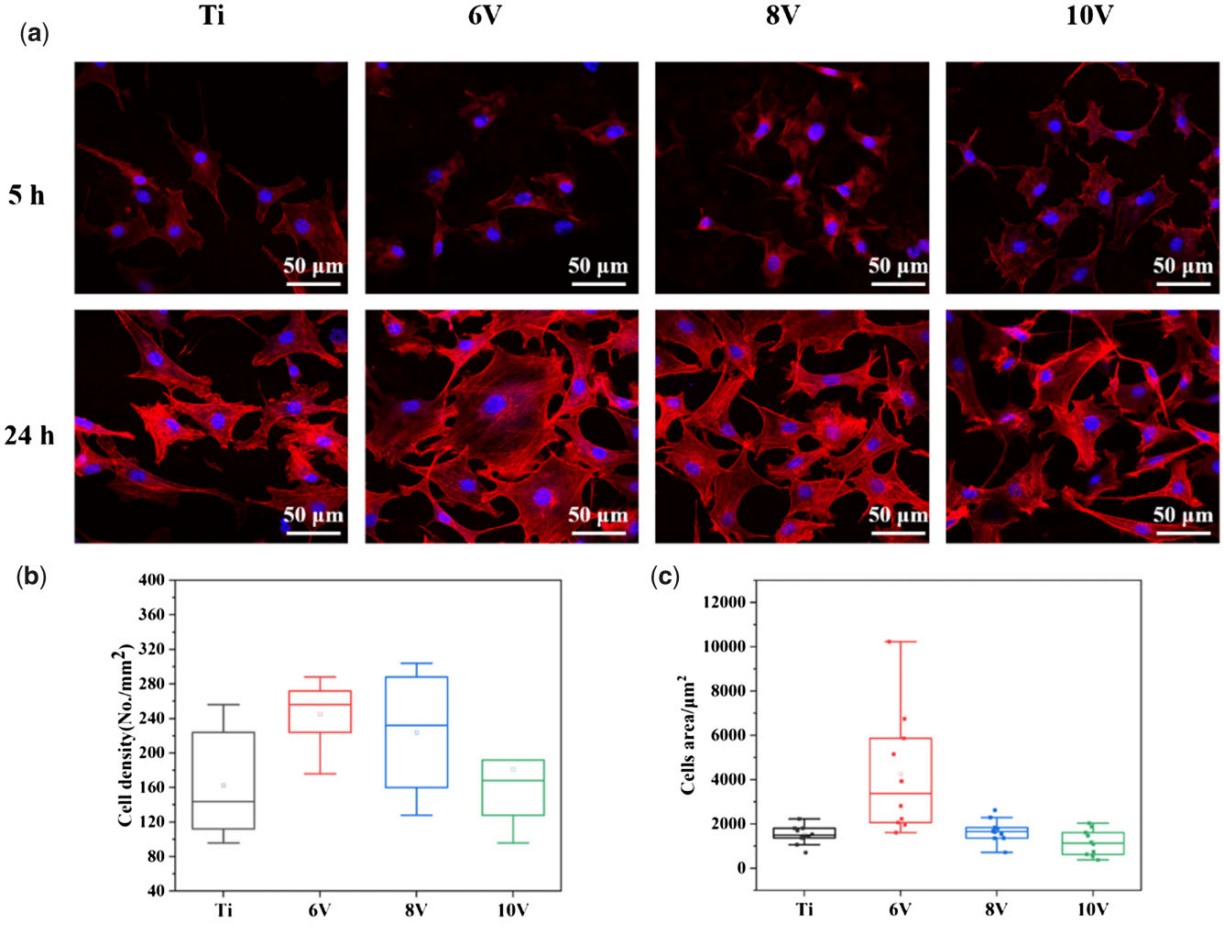
Fluorescence staining results of cytoskeleton actin and nucleus of MC3T3-E1 incubated on hierarchically structured titanium with varying micro-scaled pits for 5 h and 24 h measured by CLSM (**a**), cell density (**b**) and cells area (**c**) measured by image J software.

Subsequently, cytoskeletal actin staining was performed to analyze the specific cells adhesion status on each hierarchical sample. As shown in Fig. 7, after incubation for 5 h, the cells adhered on surface of the samples but were still not spread. The cells were small, only a few filamentous or plate-like pseudopods appeared and there was no difference of the cells spreading at the 6 V, 8 V and 10 V treated surface. When incubation time extended to 24 h, apparent actin filaments and plenty filopodia were observed on all samples, the cells began to connect to each other and stretch outwards. The cells were in the shape of fusiform or triangle on pristine titanium, while more polygonal or radial cells grown on micro/nano-titanium. The spreading areas of MC3T3-E1 on 6 V sample were obviously larger than pristine titanium, the smallest cell areas were shown on 10 V samples (Fig. 7c). Meanwhile, the quantity of cells adhered on 6 V or 8 V were higher than 10 V treated sample, which was consistent with Calcein-AM staining results (Fig. 7b). The differences of cell adhesion, spreading and growth might be caused by increased roughness or deeper micro-pits on 10 V sample, which was not conductive to the outward extension and migration of cells, thus restricted cells proliferation on this surface.

The mechanism that cells behaviors were enslaved by the materials morphology was associated with the formation of focal adhesions (FAs) and its mediated signal transmission. Cells were sensitive to the surface structure and resulting stress or strain signals could be perceived by cell membrane or intracellular receptors, then further induced cytoskeleton rearrangement [46]. The nanostructure would induce the formation of FAs, and cells migration could be mediated by formation or disappearance of FAs [28]. Meanwhile, the generation of FAs, as a vital signaling factor, would facilitate a series of cell behaviors such as cell adhesion, proliferation or differentiation through activating the relevant signaling pathways.

**Figure 8.**
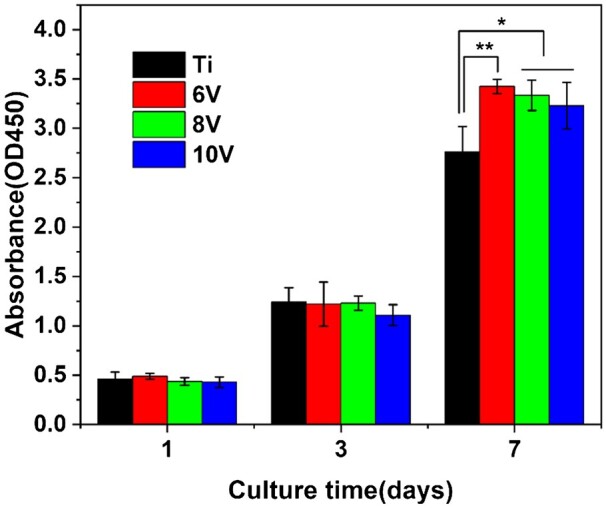
The MC3T3-E1 cell proliferation result on hierarchically structured titanium with gradient micro-scaled pits culturing for 1, 3 and 7 days. *P < 0.05 and **P < 0.01.

The result of cell proliferation on the different titanium is shown in Fig. 8. After 24-h incubation, the sample of 6 V had the most cells, and the number of cells on 10 V was the least, but there was no significant difference. When the incubation time was extended to 3 days, it was shown that little difference in the number of cells among different samples except for 10 V. The growth rate of cells on 10 V was the slowest, which probably due to greater surface roughness and depth of micro-pits are not beneficial to cell migration or proliferation [47]. It was interesting that the cells quantity on pristine titanium was the least after 7 days, due to the contact inhibition effect of cells cultured on pristine titanium with the lowest specific surface area. As for the micro–nano hierarchical titanium, larger surface area offered more space and better interfacial environment for cell proliferation, and there was great difference in cells number compared with that on pristine titanium. The trend of cells proliferation was listed as 6 V > 8V > 10V. The largest cell number on 6 V treated surface meant that the cells preferred to growth on the smaller sized microstructure. The reason might be less roughness and micron pits size on 6 V samples which facilitated cell migration and propagation.

**Figure 9.**
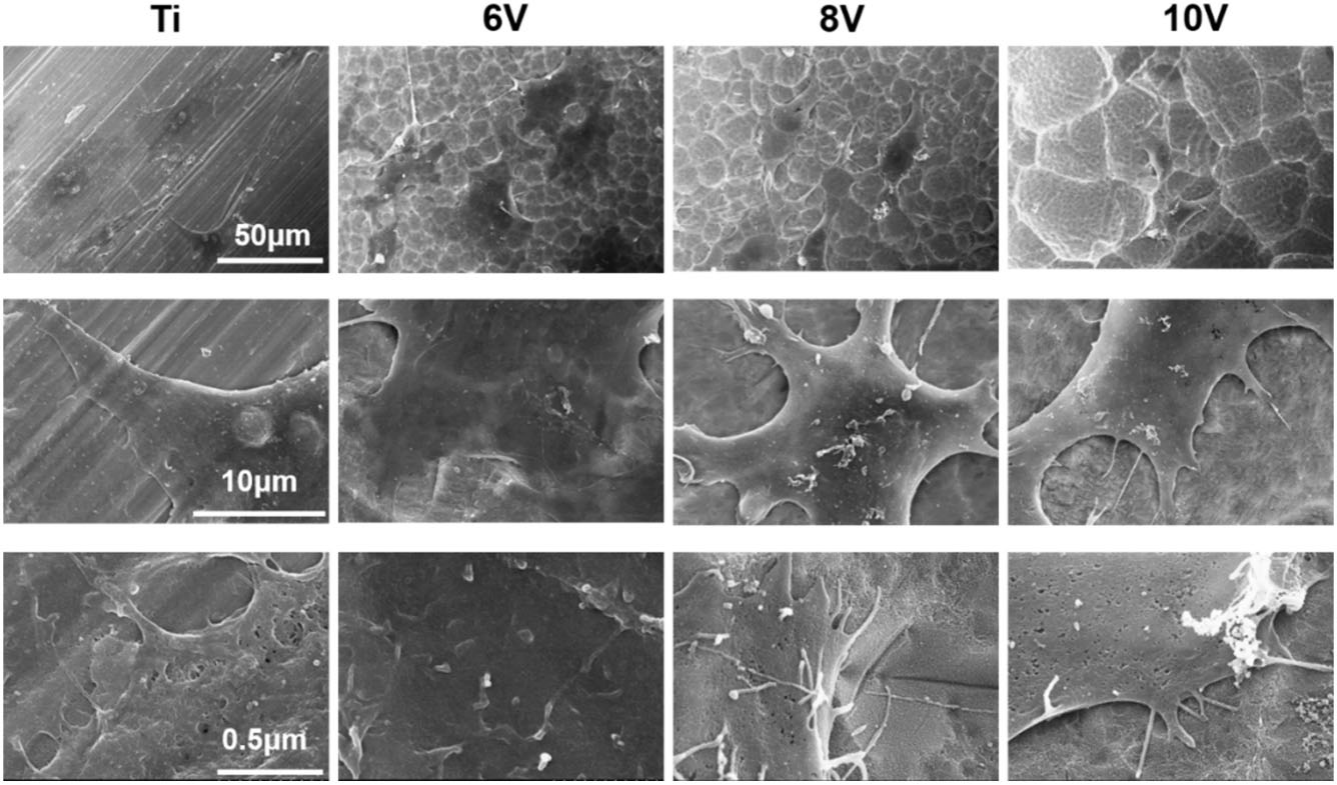
Cell morphologies cultured on hierarchically structured titanium with varying micro-scaled pits for 3 days.

**Figure 10.**
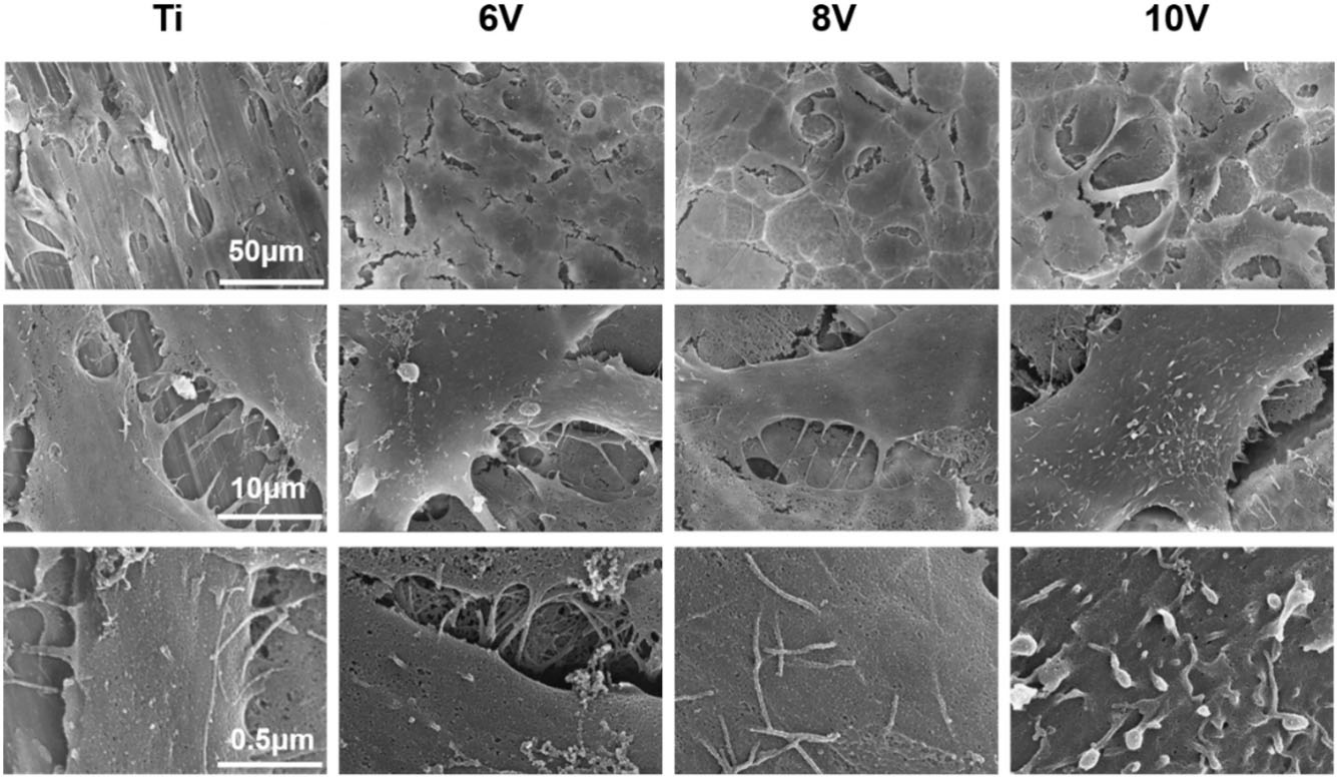
Cell morphologies cultured on hierarchically structured titanium with varying micro-scaled pits for 7 days.

It was seen that the cell could spread well on all samples, and more microvilli and filopodia were found on the ordered micro–nano hierarchical titanium compared to pristine titanium (as shown in Figs 9 and 10). The sizes of micro-pits formed at 6 V or 8 V were proved to be less than the spreading cell sizes, which gave no effect on extension and spreading of cells. At high magnification, it was found that the cell attached firmly to the substrates. While the cells anchored inside the micro-pits on the 10 V treated sample, which matched with the cellular sizes leading the cells extended outward along the edge of the micro-pits. After 7 days incubation, the cells grew well and connected to each other closely. The 6 V prepared samples presented the largest cell proliferation, where cells had covered the substrates completely, whereas some micro-pits could be observed on the 10 V samples and meant the least cell quantity. This result was consistent with WST-1 data, the rate of cell proliferation decreased with the increasing of micro-pits sizes and roughness.

### Cell osteogenic differentiation

**Figure 11.**
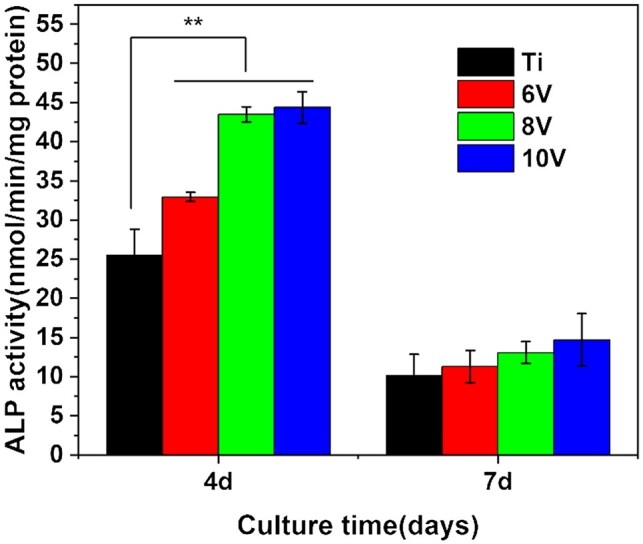
ALP activity of cells incubated on different samples for 4 and 7 days. **P < 0.01.

The ALP activity, as an indicator of cell early osteogenic differentiation, was very useful for evaluating cells osteogenic differentiation. As shown in Fig. 11, the ALP activity of MC3T3-E1 was enhanced significantly on micro–nano hierarchically structured titanium compared with that on pristine titanium after 4 days culture. While the ALP activity decreased on all samples when cells incubation time extended to 7 days. This might be attributed to that the cells stimulated by micro/nano-structure and began to differentiate toward the middle or late stage of osteogenesis with culture time extension. The overall trend of ALP activity at different samples was as follows for either 4 days or 7 days: 10 V>8V≫6V>Ti. It was indicated that micro/nano-morphology with larger scaled micro-pits was beneficial to the differentiation of cells toward osteogenesis.

**Figure 12.**
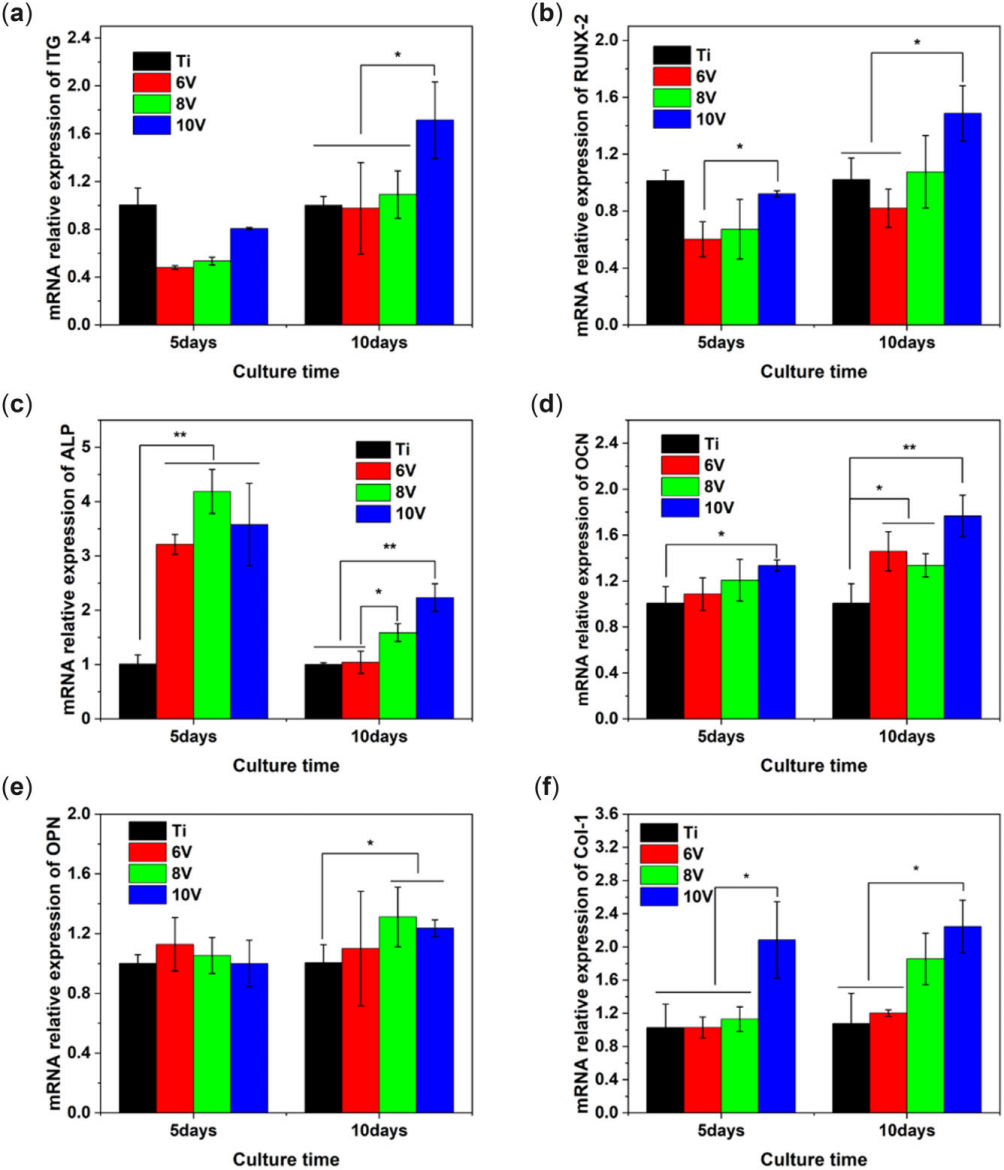
The mRNA relative expression for osteogenic genes of MC3T3-E1 cultured on the hierarchically structured titanium with varying micro-scaled pits for 5 and 10 days (**a**) ITG, (**b**) RUNX-2, (**c**) ALP, (**d**) OCN, (**e**) OPN, (**f**) Col-1. *P < 0.05 and **P < 0.01.

In order to elucidate the influence of different morphologies on osteogenic differentiation of cells, a real-time quantitative PCR was employed to detect the expression of osteogenic-specific genes for cells cultured on varying micro-scaled micro–nano multilevel structures on titanium. The integrin-related genes (ITG) played a vital media role on recognizing or binding between cells and cells or ECM, then mediated cell proliferation and differentiation as well [48]. As a DNA-binding transcription factor of Runt family, RUNX2 could activate the expression of OCN and mostly osteogenic genes, regulate cell differentiation into osteoblast and promote ECM mineralization [49]. In addition, ALP, OPN, OCN and collagen were chosen as early, middle and late differentiation indexes and detected respectively, corresponding results are shown in Fig. 12. It was suggested that the relative expressions of mRNA for ITG, RUNX-2, OCN, OPN and Col-I were in manner of positively time-dependent. The longer the incubation time, the larger the gene expression. Nevertheless, the expression of ALP presented opposite trend, the mRNA expression of cells cultured for 5 days was much higher than 10 days, and there was highly significant difference between micro/nano-titanium and pristine samples, which was in line with the results of ALP activity examined by AKP detection kit. After 10 days incubation, the expression of osteogenic-related genes were heightened obviously on micro–nano hierarchically structured titanium with larger micro-scaled pits and roughness, especially for the 10 V prepared samples. It was concluded that the expression of osteogenic genes could be adjusted and controlled distinctly by altering sizes of micro-structure, and the genes might be up-regulated on the micro–nano structured titanium with cell sized matched micro-pits.

### Wnt/β-catenin pathway

**Figure 13.**
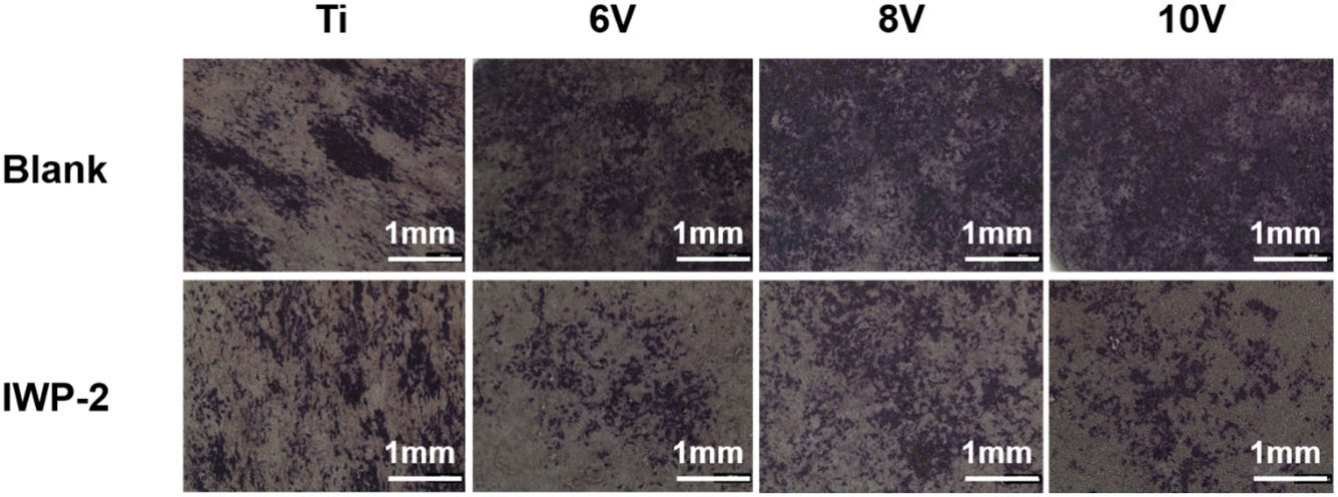
The ALP staining result for MC3T3-E1 culturing with/without Wnt/β-catenin pathway inhibitor on hierarchically structured titanium with varying micro-scaled pits.

It has been reported that the surface morphology of implants could promote bone formation through the classical Wnt/β-catenin signaling cascade [50–52]. The signal pathway of Wnt/β-catenin would be activated by combination of Wnt proteins and corresponding cell membrane receptors, then inhibited the complex composed of Axin, glycogen synthase kinase-3β and adenomatous polyposis *coli* which could reduce the degradation of β-catenin in cytoplasm. As a consequence, β-catenin accumulated and transferred into cell nucleus and subsequently reacted with transcription factor T cytokines and lymphatic enhancement factor-1 to promote the expression of genes and proteins related to osteogenesis [53]. For the sake of exploring important function of Wnt/β-catenin pathway on osteogenic response of cells cultured on the micro–nano structure with different micro-scaled micro-pits, the quantities of ALP, collagen secretion and ECM mineralization were tested by adding IWP-2 (C_22_H_18_N_4_O_2_S_3_), an inhibitor of Wnt/β-catenin pathway, into cell culture medium. IWP-2 could suppress Wnt-dependent phosphorylation of LRP6 receptor and Dvl2 and restrain the accumulation of β-catenin [54]. The staining result of ALP is shown in Fig. 13, the samples without inhibitors were used as control. It was observed that cells were colored in deep purple on control group, and more purple cells were observed on the 8 V or 10 V treated samples which meant more ALP production. While dark purple nodules decreased dramatically on all samples after inhibitor IWP-2 treatment. We assumed that Wnt/β-catenin pathway could be blocked by IWP-2, then suppressed the gene expression of ALP and reduced the production of ALP.

**Figure 14.**
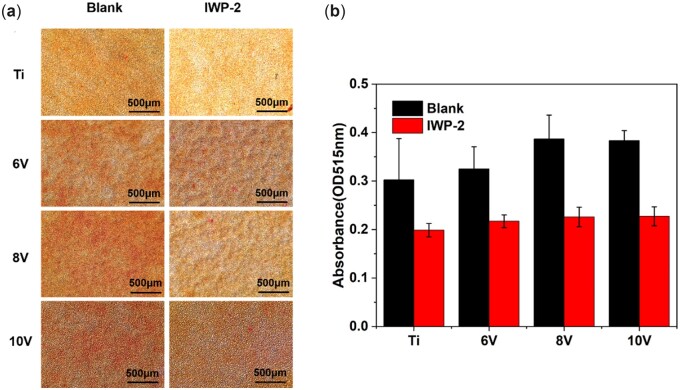
Sirius scarlet staining result of MC3T3-E1 culturing for 14 days with/without Wnt/β-catenin pathway inhibitor on hierarchical titanium with varying scaled micro-pits.

**Figure 15.**
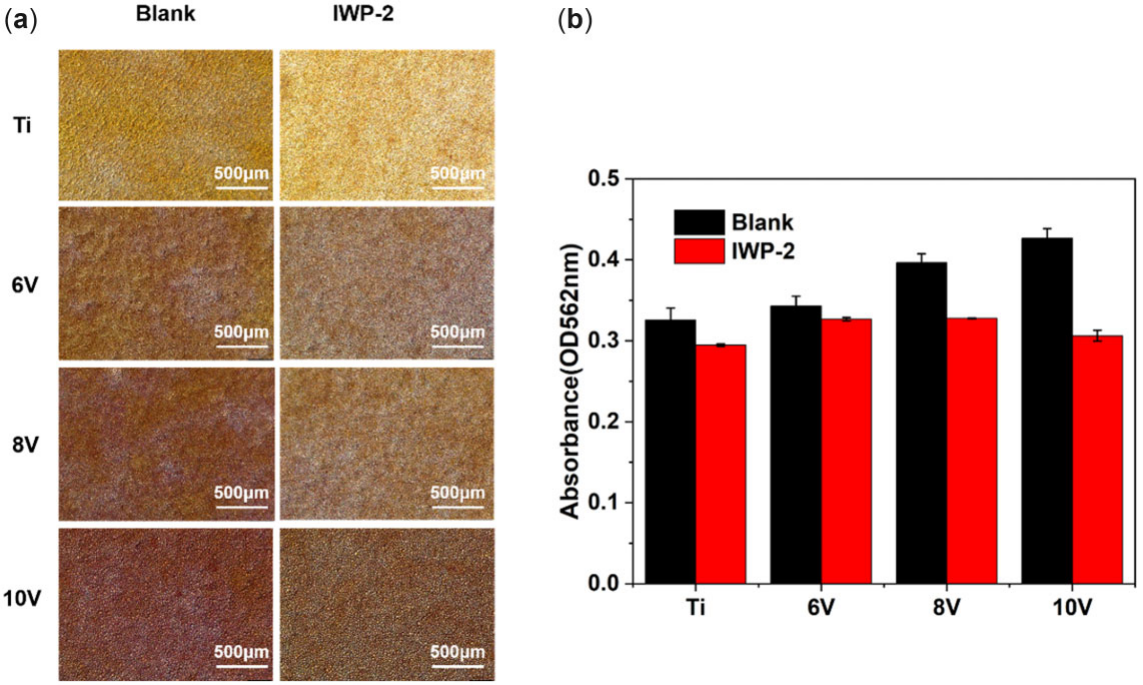
Alizarin red staining result of MC3T3-E1 culturing for 14 days with/without Wnt/β-catenin pathway inhibitor on the hierarchically structured titanium with varying scaled micro-pits.

The production of collagen was regarded as an important symbol of bone matrix formation, and the ECM mineralization was very crucial for bone maturation around implants [7]. Hence, collagen secretion and ECM mineralization of MC3T3-E1, which cultured on different micro-scaled hierarchically structured titanium with or without Wnt/β-catenin inhibitor, were also investigated. After 14-day incubation, cells were stained by Sirius red and alizarin red separately and semi-quantitative analysis were carried out by absorbance detection using microplate reader. As shown in Figs 14 and 15, cells cultured on the micro–nano hierarchically structured titanium produced much more collagen than that on pristine titanium in the absence of inhibitors. The 8 V and 10 V prepared samples exhibited larger amount of collagen, the trend for the different samples was as follows: 8 V > 10V > 6V>Ti. The reason why collagen secretion on 8 V exceeded 10 V was probably owing to the balanced development of cell proliferation and osteogenic differentiation on account of the synergistic effect of micro- or nano-structure [14]. The collagen amount on all samples decreased evidently with the addition of inhibitors. ECM could mediate cell–material interactions, and the mineralization of ECM was significance of accelerating bone forming and remolding for implants. Similarly, ECM mineralization of cells were enhanced significantly on micro–nano titanium, and the degree of mineralization was elevated with the increase of micro-pits sizes. The amount of red calcium nodules on all samples was lessened upon adding IWP-2 according to the results of alizarin red staining. It was manifested that the formation of collagen and ECM mineralization would be cut down by blocking Wnt/β-catenin signal pathway. More importantly, the vital role of Wnt/β-catenin on cells differentiation toward osteogenic mediated by micro/nano-structures was emphasized once more.

Effects of the surface microstructures of biomaterials on cell behaviors had been studied extensively. Malheiro *et al.* [55] fabricated convex and concave micro-structures on PDMS, which could change the size and shape of macrophages greatly but no effect on their polarization. In addition, the guiding roles of microstructures on cell performances for fibroblasts and bone marrow mesenchymal stem cells also been proven [56]. In order to optimize surface microstructure of orthopedic implant materials, it is very necessary to construct ordered microstructures with controlled sizes from a view of bionics. Our previous work had fabricated uniform micro-pits arrays on titanium successfully based on the electrochemical self-organization etching under constant voltage of 10 V for continuous 30 min [57], while the relationship between micro-scaled structure and cellular behaviors has not been investigated in-depth. Given the influence of current varied with applied voltage, lower constant electrochemical etching voltages were imposed on titanium to prepare various scaled micro-pits. However, the results displayed that the titanium surface could not be etched completely under voltages of 6∼9 V after 30 min. This was probably because of less corrosion pits formed on titanium at lower voltages, resulted in uneven etching. It was suggested that gradient micro-scaled pits could not be performed well only by one-step electrochemical etching. On this basis, fractional steps of electrochemical method was proposed to construct varying ordered micro-scaled pits on titanium. First, the voltage of 10 V was applied to fabricate homogeneous micro-pits due to higher interfacial field and coaction of F^–^ and Cl^–^. Then turn down the voltage to reduce the local current after 10 min, the small size micro-pits could be formed with the development and enlargement of corrosion pits. Finally, the ordered micro–nano hierarchically structured titanium with varying micro-scaled pits and identical sub-micro and nanostructure were constructed successfully, based on the combined the two-step of electrochemical etching, chemical etching and anodization. The size of micro-pits and surface roughness was enhanced with the increasing of secondary etching voltages. Meanwhile, super-hydrophilicity was presented on the multilevel structured titanium result from the presence of micro/nano structures and oxide films.

In addition, the distinct behaviors of cell adhesion and proliferation on gradient scaled micro–nano structures were closely related to the differences of micro-scale and roughness; 10 V prepared samples with cell-match-sized micro-pits array and relatively large roughness presented contrary performance on cell propagation and osteogenic differentiation. This trend was attributed to that cells growth could be limited markedly by enlarged size and depth of micro-pits. However, the Wnt/β-catenin pathway could be activated by the ordered micro–nano hierarchical morphology, and cells were induced to differentiated into osteoblasts. Certainly, the relative expression of osteogenic-specific genes, ALP activity, collagen secretion and ECM mineralization were elevated to some extent, then realized bone matures around implants eventually. Yang *et al*. [58] found that the 12 μm and 36 μm micro-dot/nanoneedle hierarchical hydroxyapatite could facilitate the polarization of macrophages to M2 phenotype, which supported that specific micro–nano hierarchical structures with larger micron scale would promote osteogenic differentiation by modulating the immune microenvironment. Moreover, Park *et al*. [56] reported that cell proliferation of fibroblasts and BMSCs on the micro-pits of 200 ∼ 300 μm would be suppressed. It was believed that intensive studying about the dependency relationship between cell behaviors and multi-scaled microstructures, as well as the corresponding molecular mechanism, could be further realized by means of advanced micro–nano processing technology and precise construction of ordered multi-scaled microstructures. Thus, this work may provide a direction and pave way to the surface design of implant materials and clinical applications. As Ding *et al.* [59, 60] pointed out that the developed micropatterning techniques are not only powerful to further understand the cell–materials interactions, but also helpful to stimulate biomaterials design and high-throughput screening in regenerative medicine.

## Conclusion

On the whole, the micro–nano hierarchically structured titanium with varying micro-scaled pits array were first fabricated by virtue of facile electrochemical self-organizing etching. The size of micro-pits array was positively correlated with the secondary etching voltage. And cells behaviors on micro/nano hierarchically structured titanium with varying micro-scaled pits has been analyzed systematically *in vitro*. It was indicated that the cells were susceptible response to micro-scaled variation and roughness changes. The cells would like to grow on micro/nano hierarchically structured titanium with smaller scaled micro-pits array. On the contrary, the larger micro-pits array was beneficial to osteogenic differentiation. It was worth mentioning that Wnt/β-catenin pathway played a crucial role on cell differentiation into osteoblast mediated by varying micro-scaled hierarchically structured titanium. This systematic study was of prominent significance for probing into the influence of micro morphology of biomaterials on cells biological performance, and provided profound strategy for surface design of implants with optimized bioactivity.

## Supplementary data

Supplementary data are available at *REGBIO* online.

## Supplementary Material

rbac046_Supplementary_Data

## References

[1] Geetha M , Singh AK, Asokamani R, Gogia AK. Ti based biomaterials, the ultimate choice for orthopaedic implants—a review. Prog Mater Sci 2009;54:397–425.

[2] Niinomi M. Biologically and mechanically biocompatible titanium alloys. Mater Trans 2008;49:2170–8.

[3] Yoon IK , Hwang JY, Jang W-C, Kim HW, Shin US. Natural bone-like biomimetic surface modification of titanium. Appl Surf Sci 2014;301:401–9.

[4] Terleeva OP , Sharkeev YP, Slonova AI, Mironov IV, Legostaeva EV, Khlusov IA, Matykina E, Skeldon P, Thompson GE. Effect of microplasma modes and electrolyte composition on micro-arc oxidation coatings on titanium for medical applications. Surf Coat Technol 2010;205:1723–9.

[5] Cheng MQ , Qiao YQ, Wang Q, Jin GD, Qin H, Zhao YC, Peng XC, Zhang XL, Liu XY. Calcium plasma implanted titanium surface with hierarchical microstructure for improving the bone formation. ACS Appl Mater Interfaces 2015;7:13053–61. 26020570 10.1021/acsami.5b03209

[6] Shen XK , Ma PP, Hu Y, Xu GQ, Zhou J, Cai KY. Mesenchymal stem cell growth behavior on micro/nano hierarchical surfaces of titanium substrates. Colloids Surf B Biointerfaces 2015;127:221–32. 25687093 10.1016/j.colsurfb.2015.01.048

[7] Kim MJ , Kim CW, Lim YJ, Heo SJ. Microrough titanium surface affects biologic response in MG63 osteoblast-like cells. J Biomed Mater Res A 2006;79:1023–32. 17034031 10.1002/jbm.a.31040

[8] Kubo K , Att W, Yamada M, Ohmi K, Tsukimura N, Suzuki T, Maeda H, Ogawa T. Microtopography of titanium suppresses osteoblastic differentiation but enhances chondroblastic differentiation of rat femoral periosteum-derived cells. J Biomed Mater Res A 2008;87:380–91. 18181115 10.1002/jbm.a.31791

[9] Boyan BD , Lossdörfer S, Wang L, Zhao G, Lohmann CH, Cochran DL, Schwartz Z. Osteoblasts generate an osteogenic microenvironment when grown on surfaces with rough microtopographies. Eur Cell Mater 2003;6:22–7. 14577052 10.22203/ecm.v006a03

[10] Lohmann CH , Bonewald LF, Sisk MA, Sylvia VL, Cochran DL, Dean DD, Boyan BD, Schwartz Z. Maturation state determines the response of osteogenic cells to surface roughness and 1,25-dihydroxyvitamin D3. J Bone Miner Res 2000;15:1169–80. 10841186 10.1359/jbmr.2000.15.6.1169

[11] Komasa S , Taguchi Y, Nishida H, Tanaka M, Kawazoe T. Bioactivity of nanostructure on titanium surface modified by chemical processing at room temperature. J Prosthodont Res 2012;56:170–7. 22613954 10.1016/j.jpor.2011.12.002

[12] Balasundaram G , Yao C, Webster TJ. TiO2 nanotubes functionalized with regions of bone morphogenetic protein-2 increases osteoblast adhesion. J Biomed Mater Res A 2008;84:447–53. 17618492 10.1002/jbm.a.31388

[13] Divya Rani VV , Manzoor K, Menon D, Selvamurugan N, Nair SV. The design of novel nanostructures on titanium by solution chemistry for an improved osteoblast response. Nanotechnology 2009;20:195101. 19420629 10.1088/0957-4484/20/19/195101

[14] Zhao LZ , Mei SL, Chu PK, Zhang YM, Wu ZF. The influence of hierarchical hybrid micro/nano-textured titanium surface with titania nanotubes on osteoblast functions. Biomaterials 2010;31:5072–82. 20362328 10.1016/j.biomaterials.2010.03.014

[15] Sugita Y , Ishizaki K, Iwasa F, Ueno T, Minamikawa H, Yamada M, Suzuki T, Ogawa T. Effects of pico-to-nanometer-thin TiO2 coating on the biological properties of microroughened titanium. Biomaterials 2011;32:8374–84. 21840046 10.1016/j.biomaterials.2011.07.077

[16] Chen XY , Cai KY, Lai M, Zhao L, Tang LL. Mesenchymal stem cells differentiation on hierarchically micro/nano-structured titanium substrates. Adv Eng Mater 2012;14:B216–B223.

[17] Zhang WJ , Wang GC, Liu Y, Zhao XB, Zou DH, Zhu C, Jin YQ, Huang QF, Sun J, Liu XY, Jiang XQ, Zreiqat H. The synergistic effect of hierarchical micro/nano-topography and bioactive ions for enhanced osseointegration. Biomaterials 2013;34:3184–95. 23380352 10.1016/j.biomaterials.2013.01.008

[18] Li GL , Cao HL, Zhang WJ, Ding X, Yang GZ, Qiao YQ, Liu XY, Jiang XQ. Enhanced osseointegration of hierarchical micro/nanotopographic titanium fabricated by microarc oxidation and electrochemical treatment. ACS Appl Mater Interfaces 2016;8:3840–52. 26789077 10.1021/acsami.5b10633

[19] Wang T , Wan Y, Liu ZQ. Effects of superimposed micro/nano-structured titanium alloy surface on cellular behaviors in vitro. Adv Eng Mater 2016;18:1259–66.

[20] Li YD , Wang WQ, Liu HY, Lei JW, Zhang JY, Zhou HZ, Qi M. Formation and in vitro/in vivo performance of “cortex-like” micro/nano-structured TiO2 coatings on titanium by micro-arc oxidation. Mater Sci Eng C Mater Biol Appl 2018;87:90–103. 29549953 10.1016/j.msec.2018.02.023

[21] Jiang PL , Zhang YM, Hu R, Wang XK, Lai YK, Rui G, Lin CJ. Hydroxyapatite-modified micro/nanostructured titania surfaces with different crystalline phases for osteoblast regulation. Bioact Mater 2021;6:1118–29. 33134605 10.1016/j.bioactmat.2020.10.006 PMC7577196

[22] Schwartz Z , Boyan BD. Underlying mechanisms at the bone-biomaterial interface. J Cell Biochem 1994;56:340–7. 7876327 10.1002/jcb.240560310

[23] Falconnet D , Csucs G, Grandin HM, Textor M. Surface engineering approaches to micropattern surfaces for cell-based assays. Biomaterials 2006;27:3044–63. 16458351 10.1016/j.biomaterials.2005.12.024

[24] Suh JY , Jang BC, Zhu XL, Ong JL, Kim K. Effect of hydrothermally treated anodic oxide films on osteoblast attachment and proliferation. Biomaterials 2003;24:347–55. 12419637 10.1016/s0142-9612(02)00325-3

[25] Salloum DS , Olenych SG, Keller TCS, Schlenoff JB. Vascular smooth muscle cells on polyelectrolyte multilayers: hydrophobicity-directed adhesion and growth. Biomacromolecules 2005;6:161–7. 15638516 10.1021/bm0497015

[26] Flemming RG , Murphy CJ, Abrams GA, Goodman SL, Nealey PF. Effects of synthetic micro- and nano-structured surfaces on cell behavior. Biomaterials 1999;20:573–88. 10213360 10.1016/s0142-9612(98)00209-9

[27] Teixeira AI , Nealey PF, Murphy CJ. Responses of human keratocytes to micro- and nanostructured substrates. J Biomed Mater Res A 2004;71:369–76. 15470741 10.1002/jbm.a.30089

[28] Mu P , Li YR, Zhang YM, Yang Y, Hu R, Zhao XL, Huang AH, Zhang RF, Liu XY, Huang QL, Lin CJ. High-throughput screening of rat mesenchymal stem cell behavior on gradient TiO2 nanotubes. ACS Biomater Sci Eng 2018;4:2804–14. 33435005 10.1021/acsbiomaterials.8b00488

[29] Song R , Zhang YM, Huang QL, Yang Y, Lin LX, Liang JH, Hu R, Rui G, Lin CJ. Facile construction of structural gradient of TiO2 nanotube arrays on medical titanium for high throughput evaluation of biocompatibility and antibacterial property. ACS Appl Bio Mater 2018;1:1056–65. 10.1021/acsabm.8b00288 34996146

[30] Kubo K , Tsukimura N, Iwasa F, Ueno T, Saruwatari L, Aita H, Chiou WA, Ogawa T. Cellular behavior on TiO2 nanonodular structures in a micro-to-nanoscale hierarchy model. Biomaterials 2009;30:5319–29. 19589591 10.1016/j.biomaterials.2009.06.021

[31] Kim HM , Miyaji F, Kokubo T, Nakamura T. Preparation of bioactive Ti and its alloys via simple chemical surface treatment. J Biomed Mater Res 1996;32:409–17. 8897146 10.1002/(SICI)1097-4636(199611)32:3<409::AID-JBM14>3.0.CO;2-B

[32] Huang QL , Elkhooly TA, Liu XJ, Zhang RR, Yang X, Shen ZJ, Feng QL. Effects of hierarchical micro/nano-topographies on the morphology, proliferation and differentiation of osteoblast-like cells. Colloids Surf B Biointerfaces 2016;145:37–45. 27137801 10.1016/j.colsurfb.2016.04.031

[33] Bai L , Du ZB, Du JJ, Yao W, Zhang JM, Weng ZM, Liu S, Zhao Y, Liu YL, Zhang XY, Huang XB, Yao XH, Crawford R, Hang RQ, Huang D, Tang B, Xiao Y. A multifaceted coating on titanium dictates osteoimmunomodulation and osteo/angio-genesis towards ameliorative osseointegration. Biomaterials 2018;162:154–69. 29454274 10.1016/j.biomaterials.2018.02.010

[34] Geng Z , Li ZY, Cui ZD, Wang J, Yang XJ, Liu CS. Novel bionic topography with MiR-21 coating for improving bone-implant integration through regulating cell adhesion and angiogenesis. Nano Lett 2020;20:7716–21. 32946240 10.1021/acs.nanolett.0c03240

[35] Ranella A , Barberoglou M, Bakogianni S, Fotakis C, Stratakis E. Tuning cell adhesion by controlling the roughness and wettability of 3D micro/nano silicon structures. Acta Biomater 2010;6:2711–20. 20080216 10.1016/j.actbio.2010.01.016

[36] Zinger O , Anselme K, Denzer A, Habersetzer P, Wieland M, Jeanfils J, Hardouin P, Landolt D. Time-dependent morphology and adhesion of osteoblastic cells on titanium model surfaces featuring scale-resolved topography. Biomaterials 2004;25:2695–711. 14962549 10.1016/j.biomaterials.2003.09.111

[37] Shin DH , Shokuhfar T, Choi CK, Lee SH, Friedrich C. Wettability changes of TiO2 nanotube surfaces. Nanotechnology 2011;22:315704. 21727317 10.1088/0957-4484/22/31/315704

[38] Lai YK , Lin LX, Pan F, Huang JY, Song R, Huang YX, Lin CJ, Fuchs H, Chi LF. Bioinspired patterning with extreme wettability contrast on TiO2 nanotube array surface: a versatile platform for biomedical applications. Small 2013;9:2945–53. 23420792 10.1002/smll.201300187

[39] Serro AP , Saramago B. Influence of sterilization on the mineralization of titanium implants induced by incubation in various biological model fluids. Biomaterials 2003;24:4749–60. 14530072 10.1016/s0142-9612(03)00372-7

[40] Lim JY , Donahue HJ. Cell sensing and response to micro- and nanostructured surfaces produced by chemical and topographic patterning. Tissue Eng 2007;13:1879–91. 17583997 10.1089/ten.2006.0154

[41] Rabe M , Verdes D, Seeger S. Understanding protein adsorption phenomena at solid surfaces. Adv Colloid Interface Sci 2011;162:87–106. 21295764 10.1016/j.cis.2010.12.007

[42] Michiardi A , Aparicio C, Ratner BD, Planell JA, Gil J. The influence of surface energy on competitive protein adsorption on oxidized NiTi surfaces. Biomaterials 2007;28:586–94. 17046057 10.1016/j.biomaterials.2006.09.040

[43] Deguchi S , Yokoyama R, Maki T, Tomita K, Osugi R, Hakamada M, Mabuchi M. A new mechanism for reduced cell adhesion: adsorption dynamics of collagen on a nanoporous gold surface. Mater Sci Eng C Mater Biol Appl 2021;119:111461. 33321592 10.1016/j.msec.2020.111461

[44] Lord MS , Foss M, Besenbacher F. Influence of nanoscale surface topography on protein adsorption and cellular response. Nano Today 2010;5:66–78.

[45] Huang QL , Lin LX, Yang Y, Hu R, Vogler EA, Lin CJ. Role of trapped air in the formation of cell-and-protein micropatterns on superhydrophobic/superhydrophilic microtemplated surfaces. Biomaterials 2012;33:8213–20. 22917736 10.1016/j.biomaterials.2012.08.017

[46] Dalby MJ , Gadegaard N, Tare R, Andar A, Riehle MO, Herzyk P, Wilkinson CDW, Oreffo ROC. The control of human mesenchymal cell differentiation using nanoscale symmetry and disorder. Nat Mater 2007;6:997–1003. 17891143 10.1038/nmat2013

[47] Faia-Torres AB , Charnley M, Goren T, Guimond-Lischer S, Rottmar M, Maniura-Weber K, Spencer ND, Reis RL, Textor M, Neves NM. Osteogenic differentiation of human mesenchymal stem cells in the absence of osteogenic supplements: a surface-roughness gradient study. Acta Biomater 2015;28:64–75. 26432440 10.1016/j.actbio.2015.09.028

[48] Xiao GZ , Wang D, Benson MD, Karsenty G, Franceschi RT. Role of the α2-integrin in osteoblast-specific gene expression and activation of the Osf2 transcription factor. J Biol Chem 1998;273:32988–94. 9830051 10.1074/jbc.273.49.32988

[49] Ducy P, Zhang R, Geoffroy V, Ridall AL, Karsenty G. Osf2/Cbfa1: a transcriptional activator of osteoblast differentiation. Cell 1997;89:747–54. 9182762 10.1016/s0092-8674(00)80257-3

[50] Wang W , Zhao LZ, Ma QL, Wang QT, Chu PK, Zhang YM. The role of the Wnt/beta-catenin pathway in the effect of implant topography on MG63 differentiation. Biomaterials 2012;33:7993–8002. 22889483 10.1016/j.biomaterials.2012.07.064

[51] Mao LX , Liu JQ, Zhao JL, Chang J, Xia LG, Jiang LY, Wang XH, Lin KL, Fang B. Effect of micro-nano-hybrid structured hydroxyapatite bioceramics on osteogenic and cementogenic differentiation of human periodontal ligament stem cell via Wnt signaling pathway. Int J Nanomedicine 2015;10:7031–44. 26648716 10.2147/IJN.S90343 PMC4648603

[52] Yu YL , Shen XK, Luo Z, Hu Y, Li MH, Ma PP, Ran QC, Dai LL, He Y, Cai KY. Osteogenesis potential of different titania nanotubes in oxidative stress microenvironment. Biomaterials 2018;167:44–57. 29554480 10.1016/j.biomaterials.2018.03.024

[53] Boyan BD , Olivares-Navarrete R, Berger MB, Hyzy SL, Schwartz Z. Role of Wnt11 during osteogenic differentiation of human mesenchymal stem cells on microstructured titanium surfaces. Sci Rep 2018;8:8588. 29872092 10.1038/s41598-018-26901-8 PMC5988747

[54] Chen BZ , Dodge ME, Tang W, Lu JM, Ma ZQ, Fan CW, Wei SG, Hao W, Kilgore J, Williams NS, Roth MG, Amatruda JF, Chen C, Lum L. Small molecule–mediated disruption of Wnt-dependent signaling in tissue regeneration and cancer. Nat Chem Biol 2009;5:100–7. 19125156 10.1038/nchembio.137 PMC2628455

[55] Malheiro V , Lehner F, Dinca V, Hoffmann P, Maniura-Weber K. Convex and concave micro-structured silicone controls the shape, but not the polarization state of human macrophages. Biomater Sci 2016;4:1562–73. 27709146 10.1039/c6bm00425c

[56] Park JY , Lee DH, Lee EJ, Lee S-H. Study of cellular behaviors on concave and convex microstructures fabricated from elastic PDMS membranes. Lab Chip 2009;9:2043–9. 19568673 10.1039/b820955c

[57] Liang J , Song R, Huang Q, Yang Y, Lin L, Zhang Y, Jiang P, Duan H, Dong X, Lin C. Electrochemical construction of a bio-inspired micro/nano-textured structure with cell-sized microhole arrays on biomedical titanium to enhance bioactivity. Electrochimica Acta 2015;174:1149–59.

[58] Yang C , Zhao CC, Wang XY, Shi MC, Zhu YL, Jing LG, Wu CT, Chang J. Stimulation of osteogenesis and angiogenesis by micro/nano hierarchical hydroxyapatite via macrophage immunomodulation. Nanoscale 2019;11:17699–708. 31545331 10.1039/c9nr05730g

[59] Yao X , Peng R, Ding JD. Cell-material interactions revealed via material techniques of surface patterning. Adv Mater 2013;25:5257–86. 24038153 10.1002/adma.201301762

[60] Yao X , Liu RL, Liang XY, Ding JD. Critical areas of proliferation of single cells on micropatterned surfaces and corresponding cell type dependence. ACS Appl Mater Interfaces 2019;11:15366–80. 30964630 10.1021/acsami.9b03780

