# Supplementary material for: Cell osteogenic bioactivity mediated precisely by varying scaled micro-pits on ordered micro/nano hierarchical structures of titanium
Source: Regen Biomater. 2022 Jul 1;9:rbac046. doi: 10.1093/rb/rbac046 (PMC9290875; doi:10.1093/rb/rbac046)
Supplement: rbac046_Supplementary_Data [file rbac046_supplementary_data.zip › SI-micro-scaled_pits.docx]

**Cell osteogenic bioactivity mediated precisely by varying scaled micro-pits on ordered micro/nano** **hierarchical structures of titanium**

Yanmei Zhang^1^, Xiankuan Wang^1^, Yaxian Li^1^, Jianhe Liang^1^, Pinliang Jiang^1^, Qiaoling Huang^2^, Yun Yang^*2^, Hongping Duan^3^, Xiang Dong^3^, Gang Rui^*4^, Changjian Lin^*1,5^

*^1^ State Key Laboratory of Physical Chemistry of Solid Surfaces, and College of Chemistry and Chemical Engineering, Xiamen University, Xiamen 361005, China;*

*^2^ Research Institute for Biomimetics and Soft Matter, Fujian Provincial Key Laboratory for Soft Functional Materials Research, College of Physical Science and Technology, Xiamen University, Xiamen 361005, China;*

*^3^Beijing Engineering Laboratory of Functional Medical Materials and Devices, Beijing Medical Implant Engineering Research Center, Beijing Naton Technology Group Co. Ltd, Beijing, China*

*^４^Department of Orthopedics Surgery, The First Affiliated Hospital of Xiamen University, Xiamen, Fujian 361003, China*

*^5^Tan Kah Kee Innovation Laboratory, Xiamen, Fujian, 361005 China*

Table S1. Primer sequences of osteogenic specific genes and reference genes

| **Primers** | **Forward** | **Reverse** |
| --- | --- | --- |
| ITG | GGTTTTGCAGCCTCTTTGGG | CGCTCCTCTGGGTTGAACAT |
| RUNX-2 | CCATAACGGTCTTCACAAATCCT | TCTGTCTGTGCCTTCTTGGTTC |
| ALP | GCACCTGCCTTACCAACTCT | GGACCTGAGCGTTGGTGTTA |
| OPN | TCCTGCGGCAAGCATTCTC | CTGCCAAACTCAGCCACTTTCA |
| OCN | GGTGCAGACCTAGCAGACACCA | AGGTAGCGCCGGAGTCTATTCA |
| Col-I | CTCCGGCTCCTGCTCCTCTTA | ACCAGGAAGTCCAGGCTGTC |
| GAPDH | GGCACAGTCAAGGCTGAGAATG | ATGGTGGTGAAGACGCCAGTA |


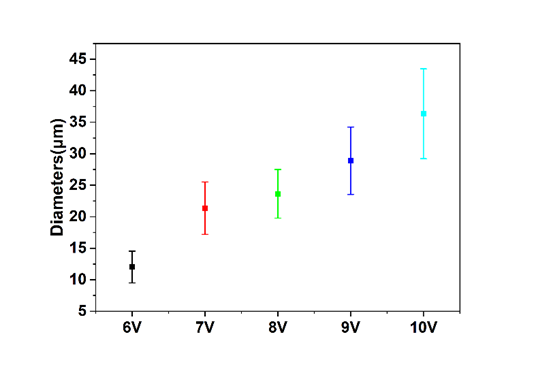


Figure S1. Diameters of varying micro-scale pits on titanium


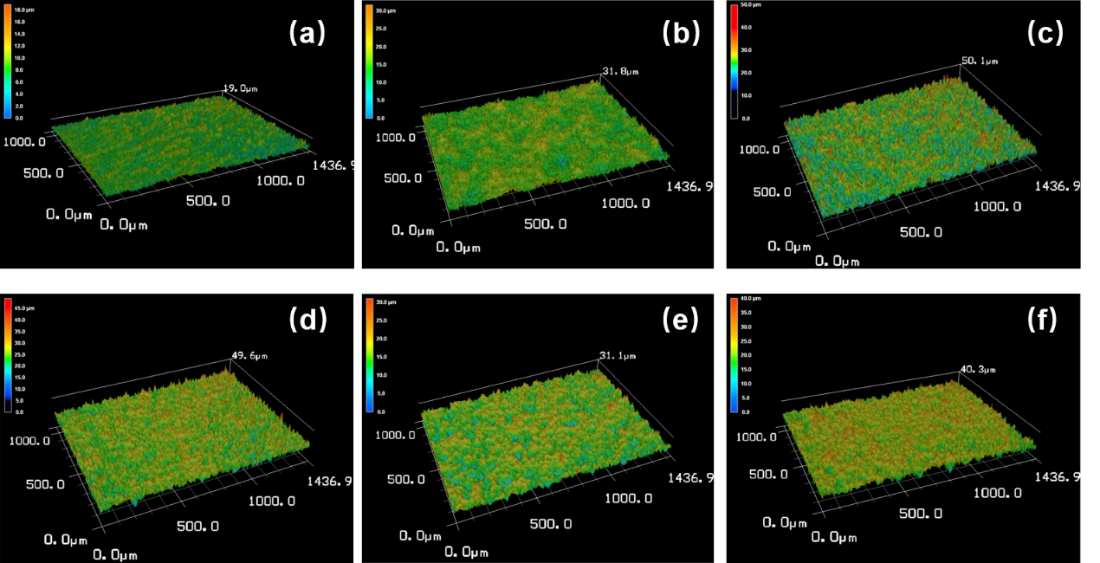


Figure S2. 3D profiles of titanium with gradient scaled micro-pits measured by laser scanning confocal microscope, (a)Ti, (b)6V, (c)7V, (d)8V, (e)9V, (f)10V.

Table S2. Roughness of different samples

|  | **Ti** | | **6V** | **7V** | **8V** | **9V** | **10V** |
| --- | --- | --- | --- | --- | --- | --- | --- |
| **Ra** | | 1.704±0.102 | 2.187±0.230 | 2.383±0.307 | 2.357±0.297 | 2.335±0.199 | 2.687±0.341 |
| **Rq** | | 2.152±0.123 | 2.830±0.314 | 3.159±0.322 | 3.030±0.305 | 2.942±0.331 | 3.395±0.357 |
| **Rz** | | 9.603±1.224 | 15.125±1.121 | 17.476±0.702 | 19.03±1.791 | 19.344±1.258 | 22.507±2.630 |
