# Supplementary material for: Cell osteogenic bioactivity mediated precisely by varying scaled micro-pits on ordered micro/nano hierarchical structures of titanium
Source: Regen Biomater. 2022 Jul 1;9:rbac046. doi: 10.1093/rb/rbac046 (PMC9290875; doi:10.1093/rb/rbac046)
Supplement: rbac046_Supplementary_Data [file rbac046_supplementary_data.zip › Response_to_reviewer_comments.docx]

**Responses to the comments from the reviewers**

**Reviewer: 1**

**Comment-1**. Most of the issues concerned by the reviewer have been well solved. Some closely related references about the cell adhesion status effect on cell proliferation and osteogenesis should be cited and discussed in relevant discussion section, such as “Cell shape, cytoskeletal tension, and RhoA regulate stem cell lineage commitment. Dev. Cell 2004; 6: 483. Critical areas of proliferation of single cells on micropatterned surfaces and corresponding cell type dependence. ACS Applied Materials & Interfaces, 2019; 11: 15366. Cell-material interactions revealed via material techniques of surface patterning. Advanced Materials, 2013; 25: 5257 etc..

**Response:** Thanks for your suggestion. Some closely related references about the cell adhesion status effect on cell proliferation and osteogenesis has be cited and discussed in relevant discussion section. At the same time, the resubmitted manuscript has been completely revised word for word and highlighted in yellow.

**Reviewer: 3**

Comments to the Author

The authors have properly addressed all concerns from the reviewers with additional experiments or reasonable explanations.

**Response:** Thank you for your kind comment.
